# Floristic Changes in the Understory Vegetation of Mixed Temperate New England Freshwater Island Forests over a Period of 33 Years

**DOI:** 10.3390/plants9111600

**Published:** 2020-11-18

**Authors:** Marjorie M. Holland, Mark Winkler

**Affiliations:** 1 Department of Biology, University of Mississippi, Oxford, MS 38677, USA; 2 2168 Winding Path Drive, Memphis, TN 38133, USA; mgw928@aol.com

**Keywords:** New Hampshire, understory vegetation, temperate island forests, species richness, Shannon diversity, *Aralia nudicaulis*, *Gaultheria procumbens*, *Gaylussacia baccata*, *Maianthemum canadense*, *Tsuga canadensis* seedlings

## Abstract

During a 33-year sampling period, we observed species richness and calculated species evenness and Shannon Diversity for understory woody seedlings and herbaceous species on three small islands in Lake Winnipesaukee, New Hampshire, and noted consistency of dominant plant species over time. Seedlings and herbaceous species were recorded and measured in 25 permanent plots that were created on the three islands in 1978. The understory species data were compiled by frequency and dominance of woody seedlings and herbaceous species. Data from 250 individual quadrats show that species richness more than doubled from 41 in 1978 to 83 species on all three islands in 2011. Species evenness on all the islands remained relatively constant in each of the four samplings. The combined Shannon’s Diversity for the three islands rose from 2.76 in 1978 to 3.37 in 2011. Dominant species in the study were *Aralia nudicaulis*, *Gaultheria procumbens*, *Gaylussacia baccata*, *Maianthemum canadense*, and *Tsuga canadensis* seedlings.

## 1. Introduction

Many of the environmental problems that challenge human society are fundamentally ecological in nature, and are threatening the sustainability of Earth′s life support systems. Recognizing these challenges, the Ecological Society of America proposed the Sustainable Biosphere Initiative (SBI) to focus on three Research Priorities: global change including the ecological causes and consequences of changes in climate; biological diversity, including natural and anthropogenic changes in patterns of genetic, species, and habitat diversity; and sustainable ecological systems, including the definition and detection of stress in natural and managed ecological systems [1]. Two months after the SBI was published, members of the international ecological community met in Mexico to recommend the International Sustainable Biosphere Initiative, noting the concept of sustainability implies the use of ecological systems (the biosphere) in a manner that satisfies current needs without compromising the needs or options of future generations [2].

**Figure 1:**
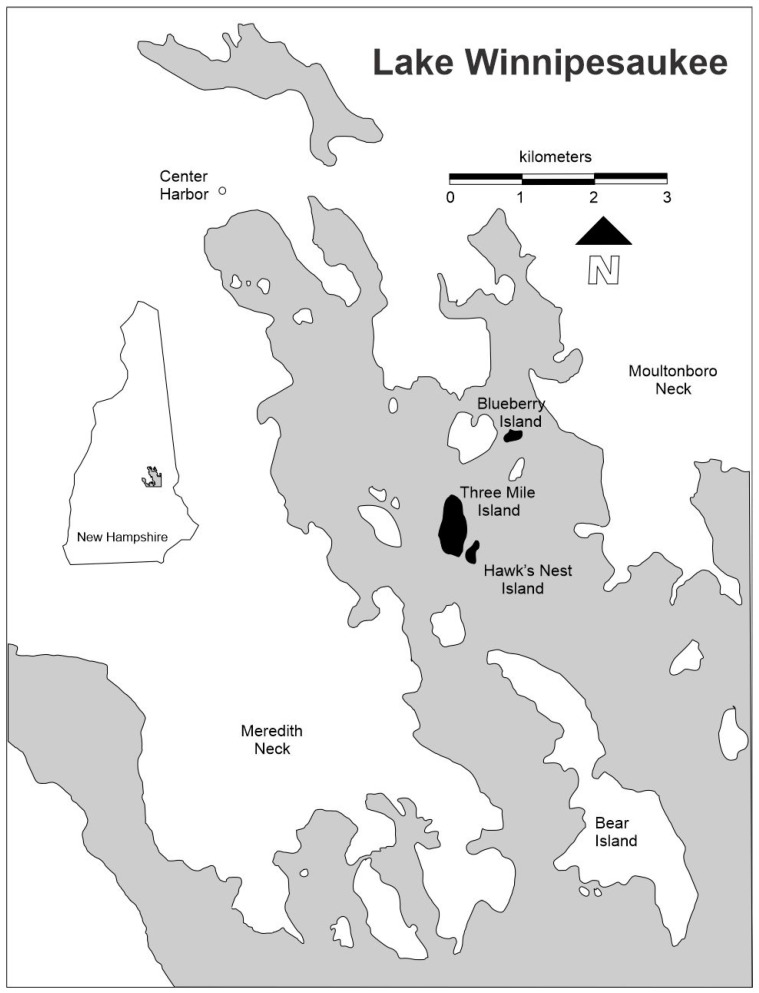
Map of the study area in northeastern Lake Winnipesaukee and location of the study sites (shown in black-shading) in New Hampshire, USA [3].

Our island studies began in the late 1970 s, and our second detailed assessment was conducted shortly after the release of the SBI report, reinforcing the need for long-term ecological assessment of biological diversity and habitat change. In this manuscript, we assess the influence of environmental variation and land-use history on forest floor plant composition and distribution on islands in central New Hampshire over 33 years. We focus on the vegetation of three of the 253 islands in Lake Winnipesaukee: Blueberry (BI), Hawk’s Nest (HNI); and Three Mile (TMI) islands (Figure 1).

These three islands are either owned or managed by the Appalachian Mountain Club (AMC), which allowed the senior author, who is a member of the Club, easy access for sampling. BI, HNI, and TMI are all within 1 km of the mainland. BI is 0.5 km from Moultonboro Neck on the mainland, TMI is 0.8 km from Meredith Neck on the mainland, and HNI is 1 km from Meredith Neck (Figure 1). Previous manuscripts present key background for these three islands: an overview of the zoning plan [3], detailed site descriptions [4], and quantitative changes in overstory vegetation [5].

TMI has an area of 17.4 hectares, HNI is 0.41 ha, and BI is 0.27 ha. All are erosional remnants formed as glaciers melted some 10,000–15,000 years ago. Because of their relative geologic youth, and the severity of the climate, soil accumulation has not been extensive [6]. Soil is deposited in till pockets estimated to be at most from five to eight feet deep, but generally, the soil is no more than one foot deep. TMI has an elevation gradient of 50 feet, with the elevation increasing further inland. Shore areas have elevations of 470 feet above sea level, while TMI’s center is at 520 feet above sea level [6]. The center of BI is 495 feet above sea level, while HNI is relatively flat, with a maximum elevation of 480 feet. The soil of all three islands is strongly acid, dominated by gravelly sand and gravelly muddy sand, low in essential nutrients, and fairly homogeneous in terms of moisture and bulk density [6].

This paper is part of a long-term study to document and quantify the flora of three islands which started in 1901, when the camp on TMI was founded, and the earliest floristic survey and plant introductions were made by landscaper Harlan P. Kelsey [7]. At the time of the 1980′s floristic survey [3], only 10 of Kelsey’s introductions remained, including *Chamaecyparis thyoides*, *Liriodendron tulipifera*, *Robinia hispida*, and *Rhododendron maximum*. By the early 1970′s the Camp had grown and the managers’ realization that certain land uses needed to be restricted led to the implementation of a land use plan [8,9]. Following an adaptation from Eugene Odum’s [10] four types of environments needed by humans, the Camp was divided into Protective, Productive, Urban, and Compromise zones [3]. The Protective zone contains unusual vegetation or natural formations (e.g., Rhododendron and Cliff Swamps); the Productive zone is managed for the harvest of firewood and wildlife habitat; the Compromise zone consists of land areas containing a small density of buildings, located along areas of shoreline; and the Urban zone consists of built-up areas and areas of heavy use. Protective and Productive zones are exposed to relatively little human use, whereas Urban and Compromise zones support the majority of human activity.

An extensive plant survey compared 1980s TMI collections [3] with collections from early 1900s [7,11], and the 1940s [12]. Species reported in the 1980s [3] which were not recorded in the early 1900s [11] include: Bog nodding aster (*Aster nemoralis*) found in the Protective Zone [least disturbed], white goosefoot (*Chenopodium album*), meadow rye grass (*Festuca elatior*), common yellow wood sorrel (*Oxalis stricta*, formerly known as *O. europaea*), reed canary grass (*Phalaris arundinacea*), and common stichwort (*Stellaria media*), all found in the Urban [most disturbed] Zone. Shade-tolerant species, such as *Lycopodium complanatum*, are typically found in the Productive Zone, while sun-tolerant species, such as *Comptonia peregrina*, are usually found in the Urban and Compromise Zones. TMI is the only island large enough (Figure 1 = map) to support an Urban Zone, with four plots near the Dining Hall, the horseshoe pit, and the Launch House designated “Urban.” The Compromise Zone (containing six plots) is along the edge of TMI, and in the center of HNI and BI. The Productive Zone also consists of six plots in the highest parts of TMI, and none occur on HNI and BI. The Protective Zone (containing nine plots) occurs in the lowest areas in the center of TMI, and on the north and south ends of HNI and BI. Recent floristic studies were also conducted on other islands in Lake Winnipesaukee, including Rattlesnake [13], Bear [14], and Timber Islands [15,16].

Islands are important in the studies of ecosystems because of their relatively “closed” habitats separated from the much larger mainland ecosystems. Ocean islands serve as individual units of ecosystems because their resident populations can be identified discretely from other habitats [17]. Studies show that introduced species [18] may have more of an effect on islands than on mainland continents. However, lake islands are not typically considered “ecological islands” as compared to oceanic islands [16]. Lake islands such as the islands of Lake Winnipesaukee are generally closer to the mainland than oceanic islands. As such, they are not as closed as oceanic islands, and their floras may be comparable to the surrounding mainland due to their close proximity and lack of definitive physical barriers that may separate their flora from the mainland flora [16].

We present results from plot-based ecological studies that quantify the frequency, distribution, and dominance of woody seedlings and understory herbaceous species listed in a 1989 floristic survey [3]. Based on our observations and knowledge of the islands in the 1970s [8,19], we predicted total species richness would be highest on the largest island which supports seven habitats (Three Mile), lowest on the smallest island with only three habitats (Blueberry), and that species richness would increase over time as parts of each island gained increased protection [20]. Second, we expected to see a decrease over time in the importance of American beech (*Fagus grandifolia*) and Eastern hemlock (*Tsuga canadensis*) seedlings, which were consumed further south by two non-native invasive species: the hemlock wooly adelgid beetle [21,22,23] and beech bark disease on the mainland [24,25]. Third, we predicted species richness would be higher in the Protective and Productive plots, and lower in the Urban and Compromise plots where most trails are located [8,20]. Fourth, we realized in 1978 as we started our sampling that we needed to educate campers and camp staff about the purpose and location of the plots, so that vegetation and habitats would not be damaged unintentionally.

## 2. Materials and Methods

Challenges encountered with long-term vegetation monitoring include: relocation of plots each decade (during each sampling the plots were photographed, and in 2011 sophisticated GPS units were used to record plot centers); plant names change over time, so names were checked each decade with the US Department of Agriculture Plant Database; field sampling records were maintained by the senior author; in 1978, personal computers were not yet available, but today options for data storage are readily available; and various software packages are available for data analyses. This study was a part of a regular ecological sampling of the vascular flora that has occurred on TMI, HNI and BI islands since 1978 [8]. The fourth sampling occurred in the summer of 2011 from June 11 to July 2 and followed the protocol of the other samplings [8,9,19]. In 1978–79, 25 permanently marked circular plots were randomly distributed across the three islands, and their numbers were assigned based on coordinates in a number grid [4,8]. Nineteen plots were established on TMI, while three plots were established on HNI, and another three on BI. Each circular plot was 34 m (111.5 ft) in diameter and was approximately 908 m^2^ [8], and the overstory plants were sampled in the circular plots [4,5,8], while 10 (1 m × 1 m) square quadrats nested within each larger plot were used to sample understory plants for a total of 250 understory plots [8,19]. Nomenclature followed respected northeastern United States taxonomic authorities [26,27].

As had been done in the 1978–79, 1991, and 2001 samplings, importance values were calculated for each understory species, using relative percent cover and relative frequency. The relative percent cover was calculated using the formula: (total percent cover of species q/total percent cover of all species) × 100. Relative frequency was calculated using the formula: (# of plots species q was found in/total # of plots of all species) × 100. The relative percent cover and relative frequency were then summed into the importance value for each species. Importance values for all species on one island total 200 (Table 1). If a zero was given as an Importance value, then that species was not present on that island. The total percent cover and total frequency for each species listed in Table 2 under “All Islands” was summed from all 250 quadrats.

Within the 250 smaller quadrats, herbaceous and woody plant species that were shorter than two meters were recorded. Nonvascular plants such as mosses were not included in this sampling. Species composition and visual estimates of percent cover for each species in each quadrat were recorded [28]. Percent covers were estimated by how much of the plot was covered by each species, and specimens were only included if the bases of their stems were found within the plot. Specimens that were overlapped by other species were included. Because of this, the total percent cover of all species may have been more than 100% in some plots. Small seedlings of specimens and small specimens were accounted for by 0.5% in the data. After all species cover was estimated, the “no vegetation” portion of the plot was estimated. This measured the portion of the plot that was not covered by any vascular plants.

Shannon’s Diversity index (Table 3) was calculated to determine the diversity of the plots and the islands. The Shannon’s Diversity index took into account both the species richness and evenness in the sampling year. The index was calculated using the following formula:

H’ = −Σ^S^_i_ = 1(p_i_*ln(p_i_)) (1)

where p_i_ is the proportion of individuals of a species (number of individuals/total # of individuals in the sampling) and S is the total number of species in the sampling. The closer the index is to ln(S), the more even the sampling. Species evenness was calculated using the formula E = H′/ln(S) where H′ is the Shannon’s Diversity Index and S is the number of species from that sample [29]. From these values, the diversity and evenness were compared across the four years of sampling for the islands’ understory.

Distributions of tree, shrub and herbaceous species were generated for each plot in each of the four land-use zones by using the bootstrap, sampling with replacement [30,31]. To provide a manageable dataset, we reported earlier on numbers of adult woody canopy species [4,5], and report on understory seedlings and herbs here. The increase in total understory species in all four zones between 1978 and 1991 was significant, and was very likely the result of a major storm in December 1980, which opened up the canopy [4]. No other significant differences in the frequency distribution of total understory species in any of the land-use zones over the periods 1991–2001 [32] and 2001–2011 [33] were observed. The 1980 storm affected eight of the sampling plots on the north end of TMI, one plot on the north end of HNI, and one plot on the northeastern end of BI. While individual trees have been struck by lightning or toppled by strong winds, no storm events as massive as the December 1980 storm have been experienced since then.

## 3. Results

### 3.1. Three Mile Island

**Figure 2:**
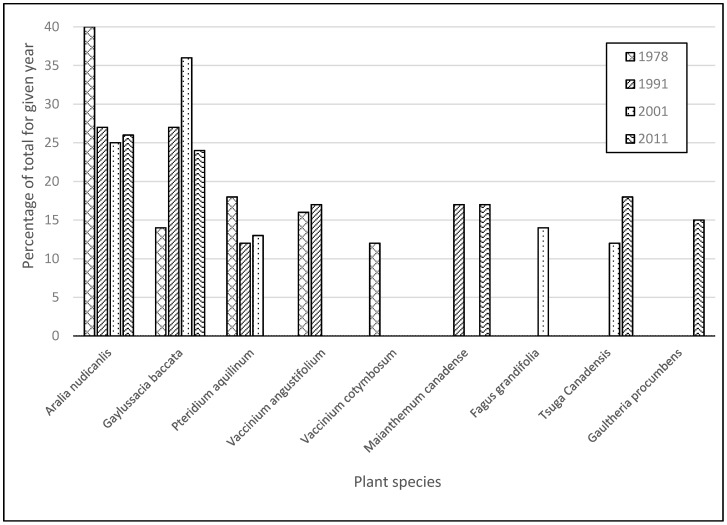
Understory dominant species by importance for all four sampling years. Values represented by percentages of the total importance value of all nine species for three islands.

Since 1978, the species with the highest importance values on TMI were (Table 1 and Table 2, Figure 2): Wild sarsaparilla (*Aralia nudicaulis*), Canada-mayflower (*Maianthemum canadense*), *Fagus grandifolia* seedlings, *Tsuga canadensis* seedlings, and black huckleberry (*Gaylussacia baccata*). By 2011, red maple (*Acer rubrum*) and *Aralia nudicaulis* appeared in all 19 plots, while striped maple (*Acer pensylvanicum*) appeared in 18 plots (Table 1). In 2011, *Aralia nudicaulis* had the highest coverage in the understory (Table 1). Species sampled on the three islands in 2011 are listed in Table 1, where importance values are included. The only “species of concern” was encountered on TMI, where bristly locust (*Robinia hispida*) was sampled in one TMI plot during 2011 [34].

### 3.2. Hawk’s Nest Island

Since 1979, the understory species with the highest importance values on Hawk’s Nest Island were (Table 1 and Table 2, Figure 2): Eastern spicy-wintergreen (*Gaultheria procumbens*), *Gaylussacia baccata*, *Tsuga canadensis* seedlings, paper birch (*Betula papyrifera*) seedlings, and *Acer rubrum* seedlings. By 2011, *Acer rubrum*, *Betula papyrifera*, and *Gaultheria procumbens*, appeared in all three sample plots (Table 1). In 2011, *Betula papyrifera* was the only birch with seedlings on the forest floor, while *Gaultheria procumbens* and *Gaylussacia baccata* had the highest coverage in the HNI plots. The center of HNI exhibited low species richness, which is likely due to its recent use for outdoor games.

### 3.3. Blueberry Island

Since 1979, the understory species with highest importance values on BI were (Table 1 and Table 2, Figure 2): *Gaylussacia baccata*, black highbush blueberry (*Vaccinium fuscatum*), common lowbush blueberry (*Vaccinium angustifolium*)*, Gaultheria procumbens*, and *Tsuga canadensis* seedlings. In 2011, the species with the highest cover on BI were *Gaylussacia baccata*, *Vaccinium fuscatum*, *Gaultheria procumbens*, *Vaccinium angustifolium*, and sheep American-laurel (*Kalmia angustifolia*) (Table 1). In 2011, *Acer rubrum* seedlings, *Tsuga canadensis* seedlings, and *Vaccinium angustifolium* appeared in all three plots (Table 1). In 2011, speckled alder (*Alnus incana* ssp. *rugosa*), *Gaylussacia baccata*, and American cow-wheat (*Melampyrym lineare*) were present in two out of the three plots on BI (Table 1).

### 3.4. Richness, Evenness, and Diversity

Over the 33-year sampling period, 130 species were sampled collectively in the three island understories (Appendix A). Throughout the sampling, evenness and diversity remained relatively constant, while species richness generally increased (Table 3).

On TMI, species richness increased significantly from 1978 to 1991 and remained relatively constant in the later samplings (Table 3). Species evenness increased from 1978 to 1991 and remained constant in the later samplings (Table 3). Furthermore, on TMI, the Shannon diversity index increased from 1978 to 1991, then remained constant throughout the next two samplings (Table 3).

On HNI, species richness increased from 15 species to 23 from 1979 to 2011 (Table 3). Species evenness and Shannon’s diversity index increased through the sampling years (Table 3). On BI, species richness increased from 8 in 1979 to 25 in 2011 (Table 3). However, species evenness fluctuated through the sampling years (Table 3). Similar to TMI, the BI Shannon diversity index remained relatively constant through the years, but it was highest during the 1991 sampling (Table 3).

Overall, the three islands saw a significant increase in total understory species richness from 1978 to 1991, though it remained relatively constant in 2001 and 2011 (Table 3). Species evenness gradually increased over the four samplings (Table 3). Lastly, Shannon’s diversity on TMI increased from 1978 to 2011, while it increased from 1979 to 2001 on HNI, then exhibited a slight decrease (Table 3). On BI, Shannon’s diversity had a noticeable increase from 1979 to 1991, dipped down in 2001, and increased in 2011. Shade-tolerant species are typically found in the Protective and Productive Zones, while sun-tolerant species are usually found in the Urban and Compromise Zones. As had been found in the overstory studies [5], approximately the same number of plant species were encountered in each of the four zones (Table 1 and Table 3).

On TMI, the following understory species were absent from the plots in 2011, but not 2001: mountain holly (*Ilex mucronata*), maleberry (*Lyonia ligustrina*), quaking poplar (*Populus tremuloides*) seedlings, eastern white oak (*Quercus alba*) seedlings, and withe-rod (*Viburnum nudum var. cassinoides*). The following species were present in the plots in 2011, but not 2001: Clinton’s wood-fern (*Dryopteris clintoniana*), three-leaved goldthread (*Coptis trifolia*), and downy rattlesnake-plantain (*Goodyera pubescens*). On HNI, highbush blueberry (*Vaccinium corymbosum*) was absent in 2011 but not 2001. The following species were present in 2011, but not 2001: Indian cucumber root (*Medeola virginiana*) and palmate hop clover (*Trifolium aureum*). On BI, the following species were absent in 2011, but not 2001: common winterberry, sweetgale (*Myrica gale*), and rhodora (*Rhododendron canadense*). The following species were present in 2011, but not 2001: eastern spicy-wintergreen, gray goldenrod (*Solidago nemoralis*), and black highbush blueberry. Even though some species were not encountered in the plots in 2011, that does not mean that they were not present in other locations outside the sample quadrats on these islands.

In a comparison of recently sampled dominant species (Table 1) with the earlier collections by J. H. Emerton, H. P. Kelsey, A. S. Pease, and R. A. Ware [3], each of the species listed as a recent dominant was present in the early 1900 s. It should be noted that while numbers of species in various plots may have increased over the 33 years of sampling, that only one new understory species, meadow rye grass (*Schedonorus pratensis*), has been recorded in the plots since the collections in the early 1900s [7,11].

## 4. Discussion

### 4.1. Succession

Since the earliest records for BI, HNI, and TMI date from the 1890s, we assume that the seeds of trees noted [35] in presettlement New Hampshire (*Fagus grandifolia, Tsuga canadensis*, and *Pinus* spp.) may have been moved by wind or mammals onto the islands. Seeds of maple, birch, and conifers are wind dispersed and with the mainland less than 1 km away from the islands (Figure 1), the mainland could have provided the seed source for these species. Records from the 1890s [36] indicate that TMI was covered by birch and poplar species, and we assume the same was true for HNI and BI, but by the early 2000s the most frequently encountered tree species seedlings were red maple, American beech, eastern white pine (*Pinus strobus*), northern red oak, and Eastern hemlock. By 2011, both paper birch and gray birch (*Betula populifolia*) seedlings were present on TMI, but were not as frequent as in the past, paper birch seedlings were present on HNI, while no birch seedlings were recorded on BI [34].

In recent years, three vistas have been maintained to allow campers to view the lake from the TMI Dining Hall. One species not recorded in the early 1900 s, but sighted in 2011, is the non-native meadow rye grass (*Schedonorus pratensis*), which was sampled in the southwestern vista. On the other hand, purple-stemmed American-aster (*Symphyotrichum puniceum* var. *puniceus*) and wavy-leaved American-aster (*Symphyotrichum undulatum*) were recorded in the early 1900 s, and were recorded again in two vistas in 2011. The native shrub, inkberry (*Ilex glabra*), was planted on TMI by H.P. Kelsey in 1901, was not recorded in TMI plots in 2011, but was sampled on the northeast end of BI in 2011. Kelsey introduced bristly locust onto TMI in 1901, and it was sampled in the southwestern vista in 1978, 1991, 2001, and 2011. Sampled in the southeastern vista in 2011 was common yellow wood sorrel.

In a study of Timber Island [16], investigators compared the flora of Timber Island to that of other Lake Winnipesaukee islands (including TMI). Application of the Simple Matching Index to the island floras of TMI and Timber Island produced a value of 65.59% [16]. This high similarity value is striking given the difference in human traffic on the two islands. Timber Island (135 acres) is the largest undeveloped island in Lake Winnipesaukee, while TMI (43 acres) experiences regular human disturbance from June through September when an AMC camp is in session. It is possible, however, that this heavy human activity has protected TMI from deer grazing in recent years [4].

Several understory species were found on TMI, HNI, BI, and Timber Island, and were sorted into frequency categories used previously [15,16]. These species include bracken fern (*Pteridium aquilinum*) (Timber: frequent; TMI: frequent; HNI: rare), eastern white pine (Timber: abundant; TMI: abundant; HNI: rare, BI: rare), eastern hemlock (Timber: abundant; TMI: frequent; HNI: occasional; BI: abundant), striped maple (Timber: occasional; TMI: abundant; HNI: rare), red maple (Timber: frequent; TMI: abundant; NHI: rare), sugar maple (*Acer saccharum*) (Timber:occasional; TMI: rare), wild sarsaparilla (Timber: rare; TMI: abundant; HNI: rare), yellow birch (*Betula alleghaniensis*) (Timber: occasional; TMI: rare), paper birch (Timber: occasional; TMI: occasional), gray birch (Timber: occasional; TMI: rare), hop-hornbeam (*Ostrya virginiana*) (Timber: frequent; TMI: occasional), eastern spicy-wintergreen (Timber: infrequent; TMI: occasional), black huckleberry (Timber: abundant; TMI: occasional), sheep American-laurel (Timber: infrequent; TMI: rare), common lowbush blueberry (Timber: infrequent; TMI: occasional), highbush blueberry (Timber: infrequent; TMI: rare), American beech (Timber: frequent; TMI: frequent), northern red oak (Timber: frequent; TMI: frequent), American witch-hazel (*Hamamelis virginiana*) (Timber: frequent; TMI; frequent), smooth shadbush (*Amelanchier laevis*) (Timber: occasional; TMI: rare; HNI: occasional), Canada-mayflower (Timber: frequent; TMI: abundant), and downy rattlesnake-plantain (*Goodyera pubescens*) (Timber: occasional; TMI: rare; NHI: rare).

### 4.2. Role of Large Grazing Herbivores

Heavy human activity is suggested to have protected TMI from deer grazing throughout the first 23 years of vegetation sampling [4], and no deer browse was observed through the 2011 sampling. However, a pregnant doe and a yearling either walked on the ice or swam over to TMI during winter 2014/spring 2015, and at least two does were sighted on numerous occasions throughout the Urban and Compromise zones during summers 2016–2019 (MMH personal observations). Thus, the senior author is concerned about the extent of deer browse on vegetation (especially on *Aralia nudicaulis, Robinia hispida*, and *Pteridium aquilium*). The upcoming 2021 sampling will provide an opportunity to quantify any herbivory noted in the long-term plots.

### 4.3. Land Use and Forest Management

This study emphasized the value of an ecological land use plan for monitoring the natural habitats of three small islands. TMI is the only one of the three islands that is large enough to support all four (Urban, Compromise, Productive, and Protective) land use zones [9]. By allowing cutting for vistas (in Urban) and clearing sick and damaged trees (to minimize risk of falling on campers in the Compromise zone), and cutting for firewood (in Productive), a balance of open and shady areas has been maintained which allows sun-tolerant and shade-tolerant understory species to survive. Both HNI and BI are zoned Protective on the north and south ends of the islands, but are zoned compromise in each island’s center (as of Autumn 2019 TMI Camp Committee minutes). Mean understory species richness in plots of all four TMI zones increased, as well as in the two zones present on HNI and BI during the 33-year sampling period (Table 3).

A recent Danish study [37] examined changes in composition and distribution of understory vegetation in two forests over a period of 23 years from 1993 to 2016. These authors note that since the beginning of the 19th century, forests in Denmark have mainly been managed as plantations with even-aged stands of one or two often-imported tree species. However, starting in the 1990s forestry practice slowly shifted towards various types of semi-natural management led by management changes in the Danish state forests [38]. Genera common to both the Danish study [37] and to our study include: *Acer* spp., *Betula* spp., *Fagus* spp., *Prunus* spp., and *Quercus* spp. seedlings; with ferns, grasses, sedges, shrubs, and forbs in the genera *Carex* spp., *Dryopteris* spp., *Galium* spp., *Oxalis* spp., *Rubus* spp., *Sambucus* spp., and *Schedonorus* spp. Species within these genera are often found in, or adjacent to, deciduous forests, where sunlight is plentiful in early spring and late summer.

### 4.4. Biodiversity and Sustainability

There is wide agreement among scientists that the future of planet Earth is at risk. Environmental problems resulting from human activities threaten the sustainability of global life-support systems [2]. Further, the earth is in the middle of a biodiversity crisis, and estimates indicate continuing and accelerating rates of global changes [39]. Scientists suggest that damaging land use and related pressures have already reduced local biodiversity intactness across 58.1% of the world’s land surface, where 71.4% of the human population live [40]. Many ecosystem services are supported by biodiversity, but globally there is currently a lack of coordinated efforts to end biodiversity declines [41].

In general, the compositions in the permanent plots of TMI, HNI, and BI demonstrated several changes in vegetation over time. During the 33-year sampling period, understory species richness increased on each island. By 2011, TMI supported more species than BI and HNI, possibly due to the success of introduced non-native species [3,7] and its larger size. However, the small BI supported more species than HNI, undoubtedly because the density of shrub growth on each end of BI allowed protection for dense understory growth. Seedlings of eastern hemlock thrived on TMI, HNI, and BI, while American beech seedlings thrived on TMI, but not on HNI nor BI [34].

Recently, authors [42] examined global sustainability of marine fisheries and noted the importance of human rewards for maintaining biodiversity. They report that getting incentives right matters and suggest that the ways in which these incentives can shift specific feedbacks in social ecological systems hold promise for conservation and management efforts in the ocean. A similar examination of incentives for management of northern forested ecosystems could prove beneficial. The Danish study [37] and the current NH study have looked at long-term management scenarios in Denmark and NH, USA, respectively, but additional long-term forest studies including incentives would be most welcome.

## 5. Conclusions

In our Introduction, we predicted total species richness would be highest on the largest island, lowest on the smallest, and that species richness would increase over time. In fact, species richness is highest on the largest island, and species richness increased over time. Lower species richness on HNI could be attributed to historical human use of the island. It appears that the proximity of freshwater islands to the mainland, along with storm effects and management strategies, may be more important in determining species richness than island size. Secondly, we expected that both American beech and Eastern hemlock would decrease in importance over time because they would be consumed by two non-native pests, but so far, Eastern hemlock is doing well on all three islands, while American beech continues to thrive on TMI. In fact, the two pests have not yet moved north to the three islands, but continue to affect hemlock and beech further south in New England. Third, the two open-canopy zones (Urban and Compromise) supported numerous sun-tolerant plant species, while the two closed-canopy zones (Protective and Productive) supported shade-tolerant species, resulting in approximately the same number of plant species growing in each of the four zones. Fourth, it appears that the land use zoning plan has encouraged campers to stay on established trails, and not wander off into the underbrush, while new construction has remained in the Urban zone. In the end, these behaviors have allowed plant species to thrive in all four zones, thus increasing species diversity overall.

## Figures and Tables

**Table 1:** Understory Importance Values for Three Mile Island (TMI), Hawk’s Nest Island (HNI), and Blueberry Island (BI) in 2011. Importance values (IV) are calculated as the sum of relative percent cover and relative frequency.

	Importance	Values	
Species	TMI	HNI	BI
*Acer pensylvanicum*	11.400	3.150	0.000
*Acer rubrum*	9.060	9.284	9.845
*Acer saccharum*	1.091	0.0	0.0
*Achillea millefolium*	0.334	0.0	0.0
*Alnus incana* ssp. *rugosa*	0.00	0.0	6.543
*Amelanchier laevis*	1.027	7.180	0.0
*Apocynum androsaemifolium*	0.00	0.0	3.061
*Aralia nudicaulis*	22.560	3.611	0.0
*Aronia melanocarpa*	0.923	6.050	0.0
*Betula alleghaniensis* var. *alleghaniensis*	1.552	0.0	0.0
*Betula papyrifera*	2.022	10.498	0.0
*Betula populifolia*	1.035	0.0	0.0
*Comptonia peregrina*	0.748	0.0	0.0
*Coptis trifolia*	0.390	0.0	0.0
*Cornus rugosa*	0.478	0.0	0.0
*Cypripedium acaule*	1.123	0.0	0.0
*Dactylis glomerata*	0.669	0.0	0.0
*Dryopteris clintoniana*	0.430	0.0	0.0
*Erigeron strigosus*	0.653	0.0	0.0
*Eurybia divaricata*	0.334	0.0	0.0
*Eurybia macrophylla*	1.218	0.0	0.0
*Euthamia graminifolia*	0.326	0.0	0.0
*Fagus grandifolia*	12.992	0.0	0.0
*Fragaria vesca* ssp. *americana*	0.581	0.0	0.0
*Gaultheria procumbens*	3.073	49.677	16.042
*Gaylussacia baccata*	11.917	29.061	39.356
*Goodyera pubescens*	0.334	3.025	0.0
*Hamamelis virginiana*	10.269	0.0	0.0
*Hieracium caespitosum*	1.035	0.0	0.0
*Ilex glabra*	0.00	0.0	3.061
*Ilex verticillata*	0.740	0.0	0.0
*Justicia americana*	0.326	7.713	0.0
*Kalmia angustifolia*	0.366	0.0	9.912
*Lindernia dubia*	0.00	0.0	3.001
*Lonicera canadensis*	1.624	0.0	0.0
*Lycopodium obscurum*	0.326	0.0	0.0
*Lyonia ligustrina*	0.00	0.0	3.482
*Lysimachia quadrifolia*	0.342	7.222	3.001
*Maianthemum canadense*	14.106	3.025	3.001
*Maianthemum racemosum* ssp. *racemosum*	1.417	0.0	0.0
*Medeola virginiana*	2.890	3.067	0.0
*Melampyrum lineare*	1.330	0.0	8.346
*Mentha arvensis*	0.326	0.0	0.0
*Mitchella repens*	3.638	3.946	0.0
*Nyssa sylvatica*	0.446	0.0	3.302
*Osmunda cinnamomea*	1.114	0.0	0.0
*Osmunda regalis*	0.334	0.0	0.0
*Ostrya virginiana*	4.323	0.0	0.0
*Oxalis stricta*	0.326	0.0	0.0
*Parthenocissus quinquefolia*	0.486	0.0	0.0
*Pinus strobus*	6.998	3.109	3.001
*Plantago major*	0.00	0.0	4.143
*Polygonatum biflorum*	0.430	0.0	0.0
*Polygonatum pubescens*	0.470	0.0	0.0
*Populus grandidentata*	0.350	0.0	0.0
*Prenanthes trifoliolata*	1.783	0.0	0.0
*Prunus serotina*	0.326	0.0	0.0
*Pteridium aquilinum*	9.983	5.034	0.0
*Quercus alba*	0.00	6.008	0.0
*Quercus rubra*	4.642	0.0	0.0
*Robinia hispida*	0.414	0.0	0.0
*Rosa palustris*	0.00	0.0	4.143
*Rubus hispidus*	0.828	0.0	0.0
*Schedonorus pratensis*	1.330	0.0	0.0
*Solidago altissima*	0.334	0.0	0.0
*Solidago caesia*	0.334	0.0	3.121
*Solidago juncea*	0.326	0.0	0.0
*Solidago nemoralis*	0.828	0.0	10.273
*Streptopus amplexifolius*	1.895	0.0	3.182
*Symphyotrichum puniceum* var. *puniceum*	0.350	0.0	0.0
*Symphyotrichum undulatum*	0.326	0.0	0.0
*Taraxacum officinale*	0.00	0.0	5.345
*Tilia Americana*	0.724	0.0	0.0
*Toxicodendron radicans*	0.462	3.192	0.0
*Trientalis borealis*	6.894	3.192	3.001
*Trifolium aureum*	0.326	3.276	0.0
*Tsuga Canadensis*	12.036	18.524	11.107
*Vaccinium angustifolium*	6.735	6.122	16.396
*Vaccinium corymbosum*	0.541	0.0	3.001
*Vaccinium fuscatum*	0.00	5.034	21.331
*Viburnum acerifolium*	7.619	0.0	0.0
*Viola blanda* var. *palustriformis*	0.342	0.0	0.0
Total	200.00	200.00	200.00

**Table 2:** Dominant Understory Species by importance values on TMI, HNI, and BI. These species are listed by decreasing importance value in [] for each island and sampling year.

	2011	2001	1991	1978
TMI	*Aralia nudicaulis* [22.6]	*Aralia nudicaulis*	*Aralia nudicaulis*	*Acer pensylvanicum*
	*Maianthemum canadense* [14.1]	*Fagus grandifolia*	*Gaylussacia baccata*	*Aralia nudicaulis*
	*Fagus grandifolia* [12.9]	*Gaylussacia baccata*	*Maianthemum canadense*	*Gaylussacia baccata*
	*Tsuga canadensis* [12.0] *Gaylussacia baccata* [11.9]	*Hamamelis virginiana*	*Pteridium aquilinum*	*Pteridium aquilinum*
		*Pteridium aquilinum*	*Vaccinium angustifolium*	*Vaccinium angustifolium*
Hawk’s	*Gaultheria procumbens* [50]	*Gaylussacia baccata*	*Gaylussacia baccata*	*Aralia nudicaulis*
Nest	*Gaylussacia baccata* [29.0]	*Pinus strobus*	*Kalmia angustifolia*	*Gaylussacia baccata*
	*Tsuga Canadensis* [19]	*Vaccinium angustifolium*	*Vaccinium angustifolium*	*Vaccinium corymbosum*
Blueberry	*Gaylussacia baccata* [39.4]	*Gaylussacia baccata*	*Gaylussacia baccata*	*Cephalanthus occidentalus*
	*Vaccinium fuscatum* [21.3]	*Ilex verticillata*	*Myrica gale*	*Vaccinium angustifolium*
	*Vaccinium angustifolium* [16.4]	*Myrica gale*	*Vaccinium angustifolium*	*Vaccinium corymbosum*
All Islands	*Aralia nudicaulis* [26]	*Gaylussacia baccata* [36]	*Aralia nudicaulis* [27]	*Aralia nudicaulis* [40]
	*Gaylussacia baccata* [24]	*Aralia nudicaulis* [25]	*Gaylussacia baccata* [27]	*Pteridium aquilinum* [18]
	*Tsuga canadensis* [18]	*Fagus grandifolia* [14]	*Maianthemum canadense* [17]	*Vaccinium angustifolium* [16]
	*Maianthemum canadense* [17]	*Pteridium aquilinum* [13]	*Vaccinium angustifolium*	*Gaylussacia baccata*
	*Gaultheria procumbens* [15]		[17]	[14]
		*Tsuga canadensis* [12]	*Pteridium aquilinum* [12]	*Vaccinium corymbosum*
				[12]

**Table 3:** Understory data compilation including species richness (Species), species evenness, and Shannon’s Diversity Index (Shannon). Data were compiled for all plots found on all islands, TMI, HNI, and BI respectively. Shannon’s Diversity Index was calculated using the EstimateS software package.

Island	Year	Species	Evenness	Shannon
All	1978	41	0.74	2.76
All	1991	81	0.74	3.27
All	2001	75	0.76	3.29
All	2011	83	0.76	3.37
Three Mile	1978	35	0.72	2.56
Three Mile	1991	69	0.74	3.15
Three Mile	2001	64	0.78	3.23
Three Mile	2011	73	0.76	3.27
Hawk’s Nest	1978	15	0.66	1.78
Hawk’s Nest	1991	20	0.67	2.00
Hawk’s Nest	2001	28	0.72	2.41
Hawk’s Nest	2011	23	0.70	2.19
Blueberry	1978	8	0.79	1.64
Blueberry	1991	27	0.80	2.64
Blueberry	2001	23	0.70	2.19
Blueberry	2011	25	0.78	2.52

## Appendix A

List of plant species by family found on TMI, HNI, and BI in any of the 25 plot samplings. Names are up to date from the United States Department of Agriculture Plant Database as of August 2020.

**Acanthaceae.**
*Justicia americana* (L.) Vahl
**Aceraceae**
*Acer pensylvanicum* L.
*Acer rubrum* L.
*Acer saccharum* Marsh.
**Anacardiaceae**
*Rhus typhina* L.
*Toxicodendron radicans* (L.) Kuntze
**Apocynaceae**
*Apocynum androsaemifolium* L.
**Aquifoliaceae**
*Ilex glabra* (L.) A. Gray
*Ilex mucronata* (L.) Powell, Savolainen & Andrews
*Ilex verticillata* (L.) A. Gray
**Araliaceae**
*Aralia nudicaulis* L.
**Asteraceae**
*Achillea millefolium* L.
*Antennaria howellii* Greene ssp. *canadensis* (Greene) Bayer
*Erigeron strigosus* Muhl. ex Willd.
*Eurybia divaricata* (L.) G. L. Nesom
*Eurybia macrophylla* (L.) Cass.
*Euthamia graminifolia* (L.) Nutt.
*Hieracium caespitosum* Dumort.
*Hieracium pilosella* L.
*Oclemena acuminata* (Michx.) Greene
*Prenanthes trifoliolata* (Cass.) Fernald
*Rudbeckia hirta* L. var. *pulcherrima* Farw.
*Solidago altissima* L.
*Solidago arguta* Aiton
*Solidago bicolor* L.
*Solidago caesia* L.
*Solidago juncea* Aiton
*Solidago nemoralis* Aiton
*Symphyotrichum lanceolatum* (Willd.) G.L. Nesom
*Symphyotrichum novi-belgii* (L.) G.L. Nesom
*Symphyotrichum puniceum* (L.) A. Love & D. Love var. puniceum
*Symphyotrichum undulatum* (L.) G.L. Nesom
*Taraxacum officinale* F.H. Wigg.
**Betulaceae**
*Alnus incana* (L.) Moench ssp. *rugosa* (Du Roi) R.T. Clausen
*Betula alleghaniensis* Britton var. *alleghaniensis*
*Betula lenta* L.
*Betula papyrifera* Marsh. var. *papyrifera*
*Betula populifolia* Marsh.
*Ostrya virginiana* (Mill.) K. Koch
**Caprifoliaceae**
*Diervilla lonicera* Mill.
*Lonicera canadensis* W. Bartram ex Marsh.
*Sambucus nigra* L. ssp. *canadensis* (L.) R. Bolli
*Viburnum acerifolium* L.
*Viburnum lentago* L.
*Viburnum nudum* L. var *cassinoides* (L.) Torr. & A. Gray
*Viburnum recognitum* Fernald
**Clusiaceae**
*Hypericum perforatum* L.
**Commelinaceae**
*Commelina communis* L.
**Cornaceae**
*Cornus rugosa* Lam.
*Nyssa sylvatica* Marsh.
**Cupressaceae**
*Juniperus communis* L. var. *depressa* Pursh
**Cyperaceae**
*Carex argyrantha* Tuck.
*Carex communis* L.H. Bailey var. *communis*
**Dennstaedtiaceae**
*Dryopteris clintoniana* (D.C. Eaton) Dowell
*Dryopteris intermedia* (Muhl. ex Willd.) A. Gray
*Dryopteris marginalis* (L.) A. Gray
*Polystichum acrostichoides* (Michx.) Schott. var. *acrostichoides*
**Ericaceae**
*Arctostaphylos uva-ursi* (L.) Spreng.
*Gaultheria procumbens* L.
*Gaylussacia baccata* (Wangenh.) K. Koch
*Kalmia angustifolia* L.
*Lyonia ligustrina* (L.) DC.
*Rhododendron canadense* (L.) Torr
*Rhododendron maximum* L.
*Vaccinium angustifolium* Aiton
*Vaccinium corymbosum* L.
*Vaccinium fuscatum* Aiton
**Fabaceae**
*Robinia hispida* L.
*Trifolium aureum* Pollich
**Fagaceae**
*Fagus grandifolia* Ehrh.
*Quercus alba* L.
*Quercus rubra* L.
**Hamamelidaceae**
*Hamamelis virginiana* L.
**Juncaceae**
*Luzula multiflora* (Ehrh.) Lej.
**Lamiaceae**
*Mentha arvensis* L.
**Liliaceae**
*Lilium philadelphicum* L.
*Maianthemum canadense* Desf.
*Maianthemum racemosum* (L.) Link ssp. *racemosum*
*Medeola virginiana* L.
*Polygonatum biflorum* (Walter) Elliott var. *biflorum*
*Polygonatum pubescens* (Willd.) Pursh
*Streptopus amplexifolius* (L.) DC.
**Lycopodiaceae**
*Dendrolycopodium obscurum* (L.) A. Haines
*Lycopodium complanatum* L.
**Monotropaceae**
*Monotropa uniflora* L.
**Myricaceae**
*Comptonia peregrina* (L.) J.M. Coult.
*Myrica gale* L.
**Oleaceae**
*Fraxinus americana* L.
*Fraxinus nigra* Marsh.
**Orchidaceae**
*Cypripedium acaule* Aiton
*Goodyera pubescens* (Willd.) R. Br.
**Orobanchaceae**
*Epifagus virginiana* (L.) W.P.C. Barton
**Osmundaceae**
*Osmunda cinnamomea* L.
*Osmunda regalis* L.
Oxalidaceae
*Oxalis stricta* L.
**Pinaceae**
*Picea rubens* Sarg.
*Pinus resinosa* Aiton
*Pinus strobus* L.
*Tsuga canadensis* (L.) Carriere
**Plantaginaceae**
*Plantago major* L.
**Poaceae**
*Dactylis glomerata* L.
*Dichanthelium boreale* (Nash) Freckmann
*Poa pratensis* L.ssp. *pratensis*
*Schedonorus pratensis* (Huds.) P. Beauv.
**Polypodiaceae**
*Polypodium virginianum* L.
**Primulaceae**
*Lysimachia quadrifolia* L.
*Trientalis borealis* Raf.
**Pyrolaceae**
*Chimaphila maculata* (L.) Pursh
**Ranunculaceae**
*Coptis trifolia* (L.) Salisb.
**Rosaceae**
*Amelanchier laevis* Wiegand
*Aronia melanocarpa* (Michx.) Elliott
*Fragaria vesca* ssp. *americana* (Porter) Staudt
*Prunus pensylvanica* L. f. var. *pensylvanica*
*Prunus serotina* Ehrh.
*Rosa palustris* Marsh.
*Rubus allegheniensis* Porter
*Rubus hispidus* L.
*Spiraea alba* Du Roi var. *latifolia* (Aiton) Dippel
**Rubiaceae**
*Cephalanthus occidentalis* L.
*Galium tinctorium* (L.) Scop.
*Mitchella repens* L.
**Salicaceae**
*Populus grandidentata* Michx.
*Populus tremuloides* Michx.
**Scrophulariaceae**
*Lindernia dubia* (L.) Pennell
*Melampyrum lineare* Desr.
**Styraceae**
*Halesia carolina* L.
**Tiliaceae**
*Tilia americana* L.
**Violaceae**
*Viola blanda* Willd. var. *palustriformis* A. Gray
*Viola renifolia* A. Gray
**Vitaceae**
*Parthenocissus quinquefolia* (L.) Planch.
